# Comparing Respiratory Illness Surveillance Case Definitions to Detect *Bordetella pertussis* in Children Aged <5 Years With Respiratory Illness in South Africa, 2017–2023

**DOI:** 10.1093/infdis/jiaf501

**Published:** 2025-10-06

**Authors:** Kate Bishop, Fahima Moosa, Mvuyo Makhasi, Jackie Kleynhans, Fathima Naby, Mignon du Plessis, Gary Reubenson, Halima Dawood, Heather J Zar, Susan Meiring, Vanessa Quan, Nicole Wolter, Anne von Gottberg, Cheryl Cohen, Alex de Voux, Sibongile Walaza

**Affiliations:** Division of Public Health Surveillance and Response, National Institute for Communicable Diseases, Division of the National Health Laboratory Service, Johannesburg; Division of Epidemiology and Biostatistics, School of Public Health, University of Cape Town, Cape Town; Centre for Respiratory Diseases and Meningitis, National Institute for Communicable Diseases, Division of the National Health Laboratory Service, Johannesburg; School of Pathology, Faculty of Health Sciences, University of the Witwatersrand, Johannesburg; Centre for Respiratory Diseases and Meningitis, National Institute for Communicable Diseases, Division of the National Health Laboratory Service, Johannesburg; School of Public Health, Faculty of Health Sciences, University of the Witwatersrand, Johannesburg; Centre for Respiratory Diseases and Meningitis, National Institute for Communicable Diseases, Division of the National Health Laboratory Service, Johannesburg; School of Public Health, Faculty of Health Sciences, University of the Witwatersrand, Johannesburg; Department of Health KwaZulu-Natal, Pietermaritzburg Metropolitan Hospitals, University of KwaZulu-Natal, Pietermaritzburg; Centre for Respiratory Diseases and Meningitis, National Institute for Communicable Diseases, Division of the National Health Laboratory Service, Johannesburg; Rahima Moosa Mother and Child Hospital, Department of Paediatrics and Child Health, School of Clinical Medicine, Faculty of Health Sciences, University of the Witwatersrand, Johannesburg; Infectious Diseases Unit, Department of Medicine, Greys Hospital, Pietermartzburg; Centre for the AIDS Programme of Research in South Africa, University of KwaZulu-Natal, Durban; Department of Paediatrics and Child Health, Red Cross War Memorial Children's Hospital, and SA-MRC Unit on Child and Adolescent Health, University of Cape Town, Cape Town; Division of Public Health Surveillance and Response, National Institute for Communicable Diseases, Division of the National Health Laboratory Service, Johannesburg; Division of Public Health Surveillance and Response, National Institute for Communicable Diseases, Division of the National Health Laboratory Service, Johannesburg; Centre for Respiratory Diseases and Meningitis, National Institute for Communicable Diseases, Division of the National Health Laboratory Service, Johannesburg; School of Pathology, Faculty of Health Sciences, University of the Witwatersrand, Johannesburg; Centre for Respiratory Diseases and Meningitis, National Institute for Communicable Diseases, Division of the National Health Laboratory Service, Johannesburg; School of Pathology, Faculty of Health Sciences, University of the Witwatersrand, Johannesburg; Division of Medical Microbiology, Department of Pathology, Faculty of Health Sciences, University of Cape Town, Cape Town; Centre for Respiratory Diseases and Meningitis, National Institute for Communicable Diseases, Division of the National Health Laboratory Service, Johannesburg; School of Public Health, Faculty of Health Sciences, University of the Witwatersrand, Johannesburg; Division of Epidemiology and Biostatistics, School of Public Health, University of Cape Town, Cape Town; Centre for Respiratory Diseases and Meningitis, National Institute for Communicable Diseases, Division of the National Health Laboratory Service, Johannesburg

**Keywords:** case definition, children, pertussis, sensitivity, surveillance

## Abstract

**Background:**

Pertussis is vaccine preventable, and surveillance can guide interventions. Assessing the performance of syndromic surveillance and the World Health Organization (WHO) pertussis case definitions can identify improvements to enhance detection and monitoring of *Bordetella pertussis.*

**Methods:**

We analyzed respiratory illness sentinel surveillance data among children aged <5 years from January 2017 through December 2023. Participants were enrolled for surveillance as outpatients with influenza-like illness (ILI) or in-patients with severe respiratory illness (SRI). Nasopharyngeal swabs were tested for *B pertussis* via polymerase chain reaction (PCR). Sensitivity and specificity, and performance indicators of case definitions were evaluated against PCR results.

**Results:**

Of 23 642 participants with PCR results, *B pertussis* was detected in 0.7% from ILI and 1.6% from SRI. When compared with the WHO pertussis case definition, a modified definition (including apnea, omitting cough duration) improved sensitivity (ILI, 30.0% vs 43.3%; SRI, 55.7% vs 60.2%) but reduced specificity (ILI, 90.5% vs 75.8%; SRI, 88.3% vs 80.9%). WHO and modified pertussis case definitions missed a large proportion of true pertussis cases (ILI, 70.0% vs 56.7%; SRI, 44.3% vs 39.8%).

**Conclusions:**

Current pertussis case definitions likely underestimate disease burden. Revising the WHO pertussis case definition and integrating pertussis into syndromic surveillance could improve detection while leveraging existing resources.

## Teaser

Pertussis surveillance case definitions showed low sensitivity in South African children aged <5 years. Revising age-specific definitions and leveraging existing respiratory surveillance resources to integrate pertussis could improve detection, provide more accurate burden estimates, support vaccine evaluation, and enable timely treatment.

*Bordetella pertussis* is a highly contagious pathogen that causes pertussis, the vaccine-preventable respiratory illness known as “whooping cough.” Global coverage with the third and final primary dose of diphtheria-tetanus-pertussis (DTP3) vaccine by 12 months of age was 84% in 2023, indicating that an estimated 14.5 million infants missed this vaccination, leaving them vulnerable to pertussis [1]. Although the Global Burden of Disease Study 2019 reported a global decline in pertussis incidence, mortality, and disability from 1990 to 2019, sub-Saharan Africa showed increasing trends, with infants aged <1 year remaining the most affected group [2, 3].

South Africa (SA) transitioned from whole cell to acellular pertussis–containing vaccine in 2009, introducing a diphtheria, tetanus, and acellular pertussis–containing combination vaccine into the national immunization program. Since then, infants receive 3 primary doses of acellular vaccine at 6, 10, and 14 weeks of age, with a booster dose at 18 months [4]. Additional booster doses at 6 and 12 years, as well as maternal vaccination in pregnancy for pertussis, were included in the vaccination schedule in 2024.

With an estimated DTP3 coverage of 82% in 2018 [5], surveillance in South African hospitals from 2013 to 2018 found a mean annual incidence of 60.7 cases per 100 000 of laboratory-confirmed *B pertussis* in children aged <5 years, with infants aged <1 year disproportionately affected (228 cases per 100 000) [6]. A nested case-control study among children in SA found a strong association between *B pertussis* and pneumonia (odds ratio [OR], 11.1; 95% CI, 1.3–92.5), while another study reported high attributable fractions (>90%) among polymerase chain reaction (PCR)–confirmed *B pertussis* cases from outpatients and hospitalized patients with respiratory illness [7, 8]. A global resurgence in pertussis has been observed since 2007, particularly in countries that transitioned to the acellular vaccine [9, 10]. Nonpharmaceutical interventions implemented during the COVID-19 pandemic disrupted the cyclical incidence of pertussis [11–13]. These factors raised concerns about waning immunity and changes in pertussis epidemiology, leading to the recommendation for additional vaccine doses in 2024. For these reasons, *B pertussis* remains a significant public health concern that requires continued surveillance to inform intervention strategies and policy making and to reduce the impact of infection.

Long-standing respiratory illness surveillance programs in SA were designed for influenza; however, the World Health Organization (WHO) currently recommends integrated surveillance of multiple respiratory pathogens through the Global Influenza Surveillance and Response System, including SARS-CoV-2 and respiratory syncytial virus, providing an opportunity to consider the usefulness of integrating pertussis [8, 14]. To provide systematic and consistent estimates of pertussis burden and trends, a highly sensitive case definition that maintains reasonable specificity would be optimal for improving the case detection of pertussis within surveillance and would be of great value in resource-limited settings where pertussis testing is not routinely performed. Increased detection could also facilitate prompt pertussis-specific treatment with macrolide antibiotics, which are not routinely given for undifferentiated respiratory illness; this would reduce infectiousness, prevent severe outcomes in infants, and limit household and community transmission through prophylactic treatment of close and vulnerable contacts [15]. In 2014, the results of a study conducted in 1 hospital in SA showed that by adapting the WHO pertussis case definition to include apnea and omit duration of cough, the sensitivity of the case definition increased [16]. This was supported by research conducted in Malaysia [17]. While the syndromic case definitions to detect other respiratory pathogens under surveillance have been evaluated [18], there remains a gap in evaluating the performance of the case definitions to detect *B pertussis* within the context of sentinel syndromic surveillance. Establishing this baseline is particularly important before evaluating the effectiveness of the recently implemented maternal pertussis vaccine. In this study, we aimed to evaluate the performance of various surveillance case definitions to detect laboratory-confirmed *B pertussis* in children aged <5 years seeking health care for respiratory illness at sentinel sites in SA.

## METHODS

### Study Design

This retrospective analysis utilized data collected prospectively by the National Institute for Communicable Diseases (NICD) between January 2017 and December 2023 from 2 prospective sentinel respiratory illness surveillance programs: influenza-like illness (ILI) at outpatient clinics and severe respiratory illness (SRI) at hospitals.

### Case Definitions

NICD's respiratory illness surveillance case definitions and the WHO pertussis case definition were evaluated for their performance in detecting *B pertussis* among children enrolled through the ILI and SRI programs against the gold standard (Table 1). The gold standard for a case of pertussis was defined as a PCR-confirmed *B pertussis* result [19] from a nasopharyngeal swab collected at the time of consultation or within 48 hours of hospital admission to exclude health care acquisition of the pathogen. The WHO severe acute respiratory infection case definition was included as an additional exploratory comparison, given its relevance in hospital-based surveillance and its frequent use in global respiratory illness studies [20, 21].

**Table 1.** Case Definitions and Year of Last Amendment for Assessment and Enrollment Into Surveillance

Case Definition: Clinical Criteria	
ILI Sentinel Surveillance: Outpatients	SRI Sentinel Surveillance: Inpatients
**NICD ILI (2022)** ^a^	**NICD SRI (2021)** ^a^
Age <5 y: acute respiratory infection with history of fever or measured fever ≥38 C° and cough with onset ≤10 d ^b^	Age <3 mo: clinician diagnosed or suspected sepsis Age <5 y: clinician-diagnosed lower respiratory tract illness
**Modified WHO pertussis (2022)** ^a^	**Modified WHO pertussis (2021)** ^a^
Age <1 y: infant with apnea Age <5 y: cough of any duration and any of the following: Paroxysms of coughing Inspiratory whoop Posttussive vomiting	Age <1 y: infant with apnea Age <5 y: cough of any duration and any of the following: Paroxysms of coughing Inspiratory whoop Posttussive vomiting
**WHO pertussis (2018)**	**WHO pertussis (2018)**
Age <1 y: cough of any duration and any of the following: Paroxysms of coughing Inspiratory whoop Posttussive vomiting	Age <1 y: cough of any duration and any of the following: Paroxysms of coughing Inspiratory whoop Posttussive vomiting
Age <5 y: cough lasting ≥14 d AND any of the following: Paroxysms of coughing Inspiratory whoop Posttussive vomiting	Age <5 y: cough lasting ≥14 d and any of the following: Paroxysms of coughing Inspiratory whoop Posttussive vomiting
	**WHO SARI (2011)**
	Age <5 y: acute respiratory infection with history of fever or measured fever ≥38 C° and cough with onset ≤10 d

Abbreviations: ILI, influenza-like illness; NICD, National Institute for Communicable Diseases; SARI, severe acute respiratory infection; SRI, severe respiratory illness; WHO, World Health Organization.

^a^Case definitions used for enrollment into surveillance and for assessment.

^b^Expansion of the ILI case definition to include COVID-19 surveillance between 2020 and 2023 including presentations with any of the following respiratory symptoms: cough, sore throat, shortness of breath, anosmia (loss of sense of smell), or dysgeusia (alteration of the sense of taste), with or without other symptoms, which may include fever, weakness, myalgia, or diarrhea.

For enrollment, 2 surveillance case definitions were applied to ILI:

*NICD ILI case definition:* an acute coughing illness (≤10 days) with either a recorded (≥38 °C) or reported history of fever

*Modified WHO pertussis case definition:* a coughing illness of any duration that presents with at least 1 of the classic pertussis symptoms (paroxysmal coughing, posttussive vomiting, inspiratory whoop); or apnea in children aged <1 year

The modified WHO pertussis case definition differs from the WHO pertussis case definition by omitting the cough duration criterion and including apnea in children aged <1 year. Surveillance systems must be adaptable, and in response to the COVID-19 pandemic, the ILI case definition was temporarily expanded between April 2020 and October 2023 to include a broader range of respiratory symptoms for COVID-19 surveillance (Table 1). Two surveillance case definitions were applied to SRI for enrollment: the NICD SRI case definition (suspected neonatal sepsis or clinician-diagnosed lower respiratory tract infection) and the modified WHO pertussis case definition.

### Recruitment and Study Population

Patients seeking health care for respiratory illness were systematically screened at sentinel sites by study-employed nurses according to established protocols for ILI and SRI. Those that met 1 or more surveillance case definitions (Table 1) and satisfied all eligibility criteria (Supplementary Table 1) were approached for informed consent from the parent or guardian.

The study population included all children aged <5 years who were enrolled through sentinel ILI or SRI surveillance and for whom PCR testing for *B pertussis* was conducted. Participants without a PCR-confirmed *B pertussis* result were excluded. A minimum sample size of 1033 was calculated per a published formula for sensitivity and specificity [22]. Input for the formula was based on previous studies in SA where the sensitivity of a modified WHO pertussis case definition was 84% and specificity was 56% [16], with an expected pertussis prevalence of 5% [23]. The confidence level was set at 95% and expected precision at 0.1.

### Study Setting and Data Collection

During the study period and across 5 provinces of SA, surveillance for ILI was conducted at 4 periurban and urban clinics, and surveillance for SRI was conducted at 8 periurban and urban hospitals (Supplementary Table 2).

Research procedures and data collection methods for the ILI and SRI protocols have been described [24, 25]. Case report forms, including demographic and clinical characteristics, underlying health conditions, vaccination status, and in-hospital outcome, were completed for all participants with ILI and SRI through structured interviews and medical record review. Nasopharyngeal swabs were collected. HIV type 1 (HIV-1) status was confirmed as part of standard of care.

### Laboratory Procedures

Nasopharyngeal swabs (Copan Italia), stored in universal transport medium (Copan Italia) at 4 to 8 °C, were transported on ice packs to the national reference laboratory for testing. Total nucleic acids were extracted via the DNA/Viral NA Small Volume extraction kit (version 2.0; Roche Diagnostics) on the MagNA Pure 96 automated extractor. *B pertussis* was detected by multiplex real-time PCR and, for the purpose of this analysis, was considered positive for *B pertussis* if the multicopy pertussis insertion sequence *481* (IS*481*) was detected with a cycle threshold (Ct) <35 or if IS*481* and *ptxS1* were detected with a Ct <40 [8, 19]. Additionally, specimens that tested positive for IS*481* were verified as negative for *Bordetella holmesii* by testing for hIS*1001*. Nasopharyngeal swabs were tested for other respiratory pathogens, including SARS-CoV-2, respiratory syncytial virus, and influenza, although only *B pertussis* results are presented in this study.

### Data Analysis

Case definition performance indicators, including sensitivity, specificity, positive predictive value (PPV), and negative predictive value (NPV), were evaluated by age group (<3 months, 3 to <12 months, 1 to <5 years). Sensitivity and specificity were arbitrarily classified as low (<50%), moderate (50%–79%), or high (≥80%). Venn diagrams illustrating the overlap between surveillance case definitions and PCR-confirmed pertussis cases were created with the DisplayR web application [26]. We used RStudio (version 2024.04.2+764) running on R (version 4.3.1; 2023-06-16 ucrt) and the UpSetR package to visualize overlaps in criteria of case definitions among PCR-confirmed pertussis cases [27–29]. Frequencies and proportions were used to describe categorical variables and were compared by χ^2^ test. For vaccination status, analyses for each primary dose of DTP vaccine included only children who were eligible to have received that dose and developed protection (≥8 weeks for a 1 primary dose, ≥12 weeks for 2 primary doses, ≥16 weeks for 3 primary doses). Children younger than the age cutoff were excluded from the dose-specific analyses. Crude ORs with 95% CIs were estimated by logistic regression to assess the association between vaccination status and PCR-confirmed *B pertussis*. Continuous variables were reported as median (IQR). Statistical tests were considered significant at *P* < .05. Data analysis was performed in Stata (version 17.0) [30].

## RESULTS

From 1 January 2017 to 31 December 2023, a total of 23 887 children aged <5 years with respiratory illness were enrolled into sentinel syndromic surveillance programs (Supplementary Figure 1). Of these, 23 642 (99.0%) had valid PCR test results for *B pertussis* and were included in the analysis: 4125 (17.4%) were outpatients enrolled through ILI surveillance and 19 517 (82.6%) were hospitalized patients through SRI surveillance.

**Figure 1.**
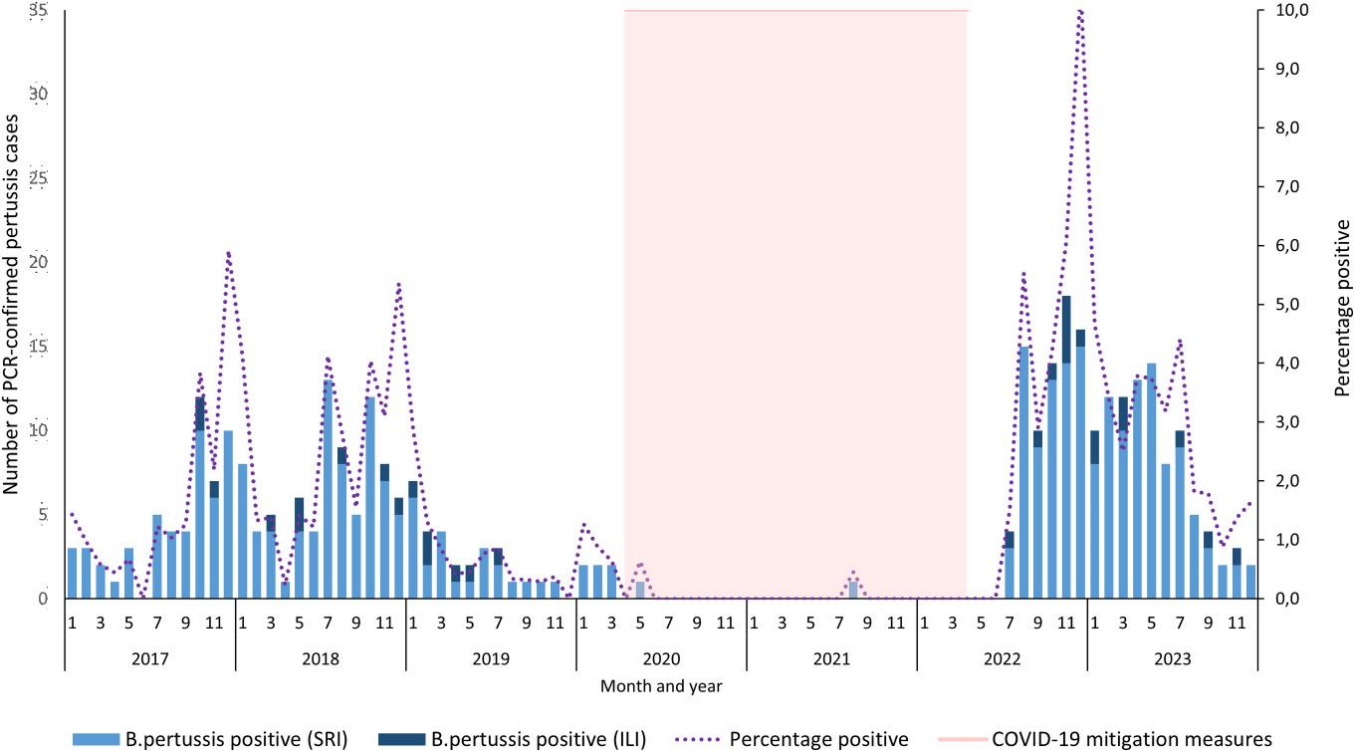
Number of sentinel surveillance *Bordetella pertussis*–positive cases confirmed by PCR in children aged <5 years by month, South Africa, 2017–2023 (n = 344): influenza-like illness (ILI) and severe respiratory illness (SRI). PCR, polymerase chain reaction.

*B pertussis* was detected among 344 of 23 642 (1.5%) participants: 30 of 4125 (0.7%) from ILI and 314 of 19 517 (1.6%) from SRI. Across the study period, the detection rate of *B pertussis* cases from both programs combined (Figure 1) had 3 distinct peaks, all occurring in December, with the highest (10.4%, 16/154) in December 2022. During the COVID-19 pandemic, 2 cases of *B pertussis* were detected between April 2020 and April 2022 when pandemic mitigation measures were most stringent.

**Figure 2.**
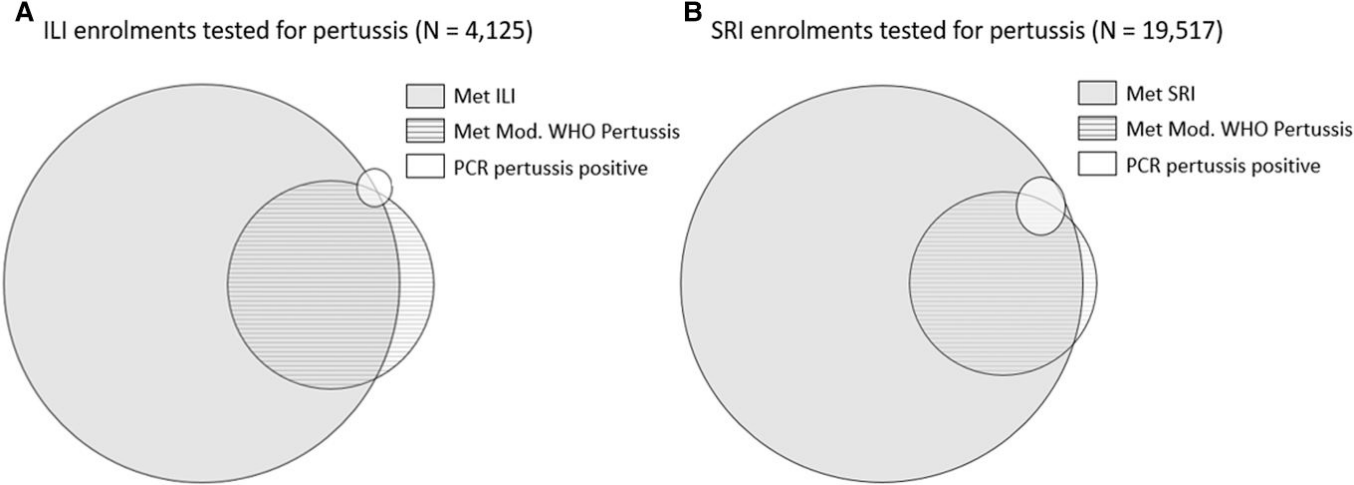
Proportional Venn diagram shows the intersection of respiratory syndromic surveillance case definitions and laboratory-confirmed PCR-positive pertussis cases among all children aged <5 years with (*A*) ILI and (*B*) SRI and a laboratory-confirmed pertussis result. Where PCR-positive pertussis cases do not overlap with ILI or modified WHO pertussis, such cases were enrolled during the COVID-19 pandemic when the ILI case definition was temporarily expanded to include a broader range of COVID-19–associated symptoms, as described in the methods. ILI, influenza-like illness; Mod, modified; PCR, polymerase chain reaction; SRI, severe respiratory illness; WHO, World Health Organization.

Among participants with ILI (n = 4125), 3731 (90.4%) met the NICD ILI case definition, and 1005 (24.4%) met the modified WHO pertussis case definition. Of the 30 PCR-positive *B pertussis* cases in ILI, 19 (63.3%) met the NICD ILI case definition, and 13 (43.3%) met the modified WHO pertussis case definition, with 2 (6.7%) meeting both definitions (Figure 2*A*). Notably, 4 (13.3%) children who were PCR positive met neither case definition. These cases were enrolled during the COVID-19 pandemic period when the ILI case definition was temporarily expanded to include a broader range of COVID-19–associated symptoms. Among participants with SRI (n = 19 517), 18 356 (94.1%) met the NICD SRI case definition, and 3851 (19.7%) met the modified WHO pertussis case definition. Of the 314 PCR-positive *B pertussis* cases in the SRI surveillance, 306 (97.4%) met the NICD SRI case definition, and 189 (60.2%) met the modified WHO pertussis case definition, with 184 (58.6%) meeting both definitions (Figure 2*B*).

When characteristics of participants who were *B pertussis* positive vs negative were compared (Supplementary Table 3), children who were pertussis positive were younger in ILI (median, 6.2 months [IQR, 2.1–15.8] vs 19.9 [9.0–37]) and SRI (1.7 months [IQR, 1.2–2.4] vs 6.3 [2.0–16.3]), with the majority aged <3 months (40.0% in ILI, 81.2% in SRI). Symptom duration at presentation was also longer among children who were pertussis positive (ILI, 5 days [IQR, 2–8] vs 3 [2–4]; SRI, 3 days [IQR, 2–7] vs 2 [1–3]). Classic pertussis symptoms were more common among positive cases: paroxysmal cough (36.7% vs 16.2% in ILI, 43.0% vs 10.9% in SRI), inspiratory whoop (13.3% vs 2.7% in ILI, 15.6% vs 2.0% in SRI), and posttussive vomiting in SRI (32.8% vs 10.7%). Apnea was also more frequent among children aged <1 year with pertussis (4.8% vs 0.4% in ILI, 17.8% vs 4.2% in SRI). However, fever was less common among participants who were pertussis positive than negative (73.3% vs 93.8% in ILI, 47.1% vs 65.2% in SRI). Among infants old enough to have completed DTP3 and developed protection (≥16 weeks of age), vaccine coverage was lower among *B pertussis*–positive cases as compared with negative ones (ILI, 54.2% vs 85.4%; SRI, 8.3% vs 52.9%), and receipt of DTP3 was associated with lower odds of PCR-confirmed *B pertussis* in ILI (crude OR, 0.14; 95% CI, .06–.36) and SRI (crude OR, 0.07; 95% CI, .05–.11).

In ILI (Table 2) and SRI (Table 3) surveillance, the NICD respiratory illness case definitions had the highest sensitivity (ILI, 63.3% [95% CI, 43.9%–80.1%], moderate; SRI, 97.5% [95% CI, 95.0%–98.9%], high) but lowest specificity for all age groups (ILI, 9.3% [95% CI, 8.5%–10.3%], low; SRI, 6.0% [95% CI, 5.7%–6.4%], low), detecting most PCR-confirmed pertussis cases while identifying many children without pertussis. However, the WHO pertussis case definition was highly specific (ILI, 90.5% [95% CI, 89.6%–91.4%]; SRI, 88.3% [95% CI, 87.8%–88.7%]) but had low to moderate sensitivity for all age groups (ILI, 30.0% [95% CI, 14.7%–49.4%], low; SRI, 55.7% [95% CI, 50.0%–61.3%], moderate), missing many PCR-confirmed cases (ILI, 70.0%; SRI, 44.3%; Table 4). The modified WHO pertussis case definition had a low to moderate sensitivity (ILI, 43.3% [95% CI, 25.5%–62.6%], low; SRI, 60.2% [95% CI, 54.5%–65.6%], moderate) and moderate to high specificity (ILI, 75.8% [95% CI, 74.4%–77.1%], moderate; SRI, 80.9% [95% CI, 80.4%–81.5%], high) but also missed many PCR-confirmed cases (ILI, 56.7%; SRI, 39.8%). The WHO and modified WHO pertussis case definitions performed best in the youngest group (<3 months), although sensitivity declined progressively with the older age groups (3 to <12 months and 1 to <5 years). PPVs were low across ILI and SRI, with high proportions of false positives (>90%) identified for all applied case definitions, while NPVs were high, indicating that most children not meeting the definitions truly did not have PCR-confirmed pertussis.

**Table 2.** Performance of Case Definitions for Detecting Laboratory-Confirmed *Bordetella pertussis* by Age Group in Outpatients With ILI at Sentinel Surveillance Sites, South Africa, 2017–2023

	Cases		Sensitivity		Specificity		PPV^a^		NPV^b^	
Age Group: Case Definition	No.	%	%	95% CI	%	95% CI	%	95% CI	%	95% CI
<3 m (n = 262)										
Gold standard PCR positive	12	4.6								
NICD ILI			58.3	27.7–84.8	10.0	6.6–14.5	3.0	1.2–6.1	83.3	65.3–94.4
Modified WHO pertussis			50.0	21.1–78.9	75.9	70.1–81.1	9.1	3.4–18.7	96.9	93.4–98.9
WHO pertussis			50.0	21.1–78.9	75.9	70.1–81.1	9.1	3.4–18.7	96.9	93.4–98.9
3–11 mo (n = 1167)										
Gold standard PCR positive	9	0.8								
NICD ILI			55.6	21.2–86.3	8.2	6.7–9.9	0.5	.2–1.1	96.0	90.0–98.9
Modified WHO pertussis			33.3	7.5–70.1	73.1	70.5–75.7	1.0	.2–2.8	99.3	98.5–99.7
WHO pertussis			33.3	7.5–70.1	73.3	70.6–75.8	1.0	.2–2.8	99.3	98.5–99.7
1–4 y (n = 2694)										
Gold standard PCR positive	9	0.3								
NICD ILI			77.8	40.0–97.2	9.8	8.7–11.0	0.3	.1–0.6	99.2	97.3–99.9
Modified WHO pertussis			44.4	13.7–78.8	76.9	75.3–78.5	0.6	.2–1.6	99.8	99.4–99.9
WHO pertussis			0.0	0–33.6	99.3	98.9–99.6	0.0	0–17.6	99.7	99.4–99.9
<5 y (n = 4123)										
Gold standard PCR positive	30	0.7								
NICD ILI			63.3	43.9–80.1	9.3	8.5–10.3	0.5	.3–.8	97.2	95.0–98.6
Modified WHO pertussis			43.3	25.5–62.6	75.8	74.4–77.1	1.3	.7–2.2	99.5	99.1–99.7
WHO pertussis			30.0	14.7–49.4	90.5	89.6–91.4	2.3	1.0–4.3	99.4	99.1–99.7

Refer to Table 1 for the clinical criteria of case definitions.

Abbreviations: ILI, influenza-like illness; NICD, National Institute for Communicable Diseases; NPV, negative predictive value; PCR, polymerase chain reaction; PPV, positive predictive value; WHO, World Health Organization.

^a^PPV: probability that a child meeting the case definition is PCR positive.

^b^NPV: probability that a child not meeting case definition is PCR negative.

**Table 3.** Performance of Case Definitions for Detecting Laboratory-Confirmed *Bordetella pertussis* by Age Group in Hospitalized Patients With SRI at Sentinel Surveillance Sites, South Africa, 2017–2023

	Cases		Sensitivity		Specificity		PPV^a^		NPV^b^	
Age Group: Case Definition	No.	%	%	95% CI	%	95% CI	%	95% CI	%	95% CI
<3 mo (n = 262)										
Gold standard PCR positive	255	3.8								
NICD SRI			98.0	95.5–99.4	6.4	5.8–7.0	4.0	3.5–4.5	98.8	97.2–99.6
WHO SARI			40.0	33.9–46.3	61.7	60.5–62.9	4.0	3.3–4.8	96.3	95.7–96.8
Modified WHO pertussis			63.9	57.7–69.8	81.3	80.3–82.2	11.9	10.3–13.8	98.3	97.9–98.6
WHO pertussis			60.8	54.5–66.8	85.9	85.1–86.8	14.7	12.6–16.9	98.2	97.8–98.5
3–11 mo (n = 1167)										
Gold standard PCR positive	38	0.6								
NICD SRI			92.1	78.6–98.3	7.7	7.1–8.4	0.6	0.4–0.8	99.4	98.3–99.9
WHO SARI			55.3	38.3–71.4	39.6	38.4–40.8	0.5	0.3–0.8	99.3	98.9–99.6
Modified WHO pertussis			50.0	33.4–66.6	78.9	77.9–79.9	1.4	0.8–2.1	99.6	99.4–99.8
WHO pertussis			47.4	31.0–64.2	80.0	79.0–81.0	1.4	0.8–2.2	99.6	99.4–99.8
1–4 y (n = 2694)										
Gold standard PCR positive	21	0.3								
NICD SRI			100.0	83.9–100.0	3.9	3.4–4.4	0.3	0.2–0.5	100.0	98.5–100.0
WHO SARI			47.6	25.7–470.2	31.5	30.4–32.7	0.2	0.1–04	99.5	99.0–99.7
Modified WHO pertussis			33.3	14.6–57.9	82.7	81.7–83.6	0.6	0.3–1.3	99.7	99.6–99.9
WHO pertussis			9.5	1.2–30.4	99.1	98.8–99.3	3.3	0.4–11.3	99.7	99.5–99.8
<5 y (n = 4123)										
Gold standard	314	1.6								
NICD SRI			97.5	95.0–98.9	6.0	5.7–6.4	1.7	1.5–1.9	99.3	98.6–99.7
WHO SARI			42.4	36.8–48.0	44.3	43.6–45.0	1.2	1.0–1.5	97.9	97.6–98.2
Modified WHO pertussis			60.2	54.5–65.6	80.9	80.4–81.5	4.9	4.2–5.6	99.2	99.1–99.3
WHO pertussis			55.7	50.0–61.3	88.3	87.8–88.7	7.2	6.2–8.3	99.2	99.0–99.3

Abbreviations: NICD, National Institute for Communicable Diseases; NPV, negative predictive value; PCR, polymerase chain reaction; PPV, positive predictive value; SARI, severe acute respiratory infection; SRI, severe respiratory illness; WHO, World Health Organization.

Refer to Table 1 for the clinical criteria of case definitions.

^a^PPV: probability that a child meeting the case definition is PCR positive.

^b^NPV: probability that a child not meeting case definition is PCR negative.

**Table 4.** Proportions of Laboratory-Confirmed *Bordetella pertussis*–Positive Cases Missed by Surveillance Case Definition per Age Group in Outpatients With ILI and Hospitalized Patients With SRI at Sentinel Surveillance Sites, South Africa, 2017–2023

ILI Surveillance (n = 30)			SRI Surveillance (n = 314)		
Case Definition	Cases Missed, %	No.	Case Definition	Cases Missed, %	No.
**Age <3 mo**					
			WHO SARI	60.0	153
NICD ILI	41.7	5	SRI	2.0	5
Modified WHO pertussis	50.0	6	Modified WHO pertussis	36.1	92
WHO pertussis	50.0	6	WHO pertussis	39.2	100
**Age, 3–11 mo**					
			WHO SARI	44.7	17
NICD ILI	44.4	4	SRI	7.9	3
Modified WHO pertussis	66.7	6	Modified WHO pertussis	50.0	19
WHO pertussis	66.7	6	WHO pertussis	52.6	20
**Age, 1–4 y**					
			WHO SARI	52.4	11
NICD ILI	22.2	2	SRI	0.0	0
Modified WHO pertussis	55.6	5	Modified WHO pertussis	66.7	14
WHO pertussis	100.0	9	WHO pertussis	90.5	19
**Age <5 y**					
			WHO SARI	57.6	181
NICD ILI	36.7	11	SRI	2.5	8
Modified WHO pertussis	56.7	17	Modified WHO pertussis	39.8	125
WHO pertussis	70.0	21	WHO pertussis	44.3	139

Refer to Table 1 for the clinical criteria of case definitions.

Abbreviations: ILI, influenza-like illness; NICD, National Institute for Communicable Diseases; SARI, severe acute respiratory infection; SRI, severe respiratory illness; WHO, World Health Organization.

**Figure 3.**
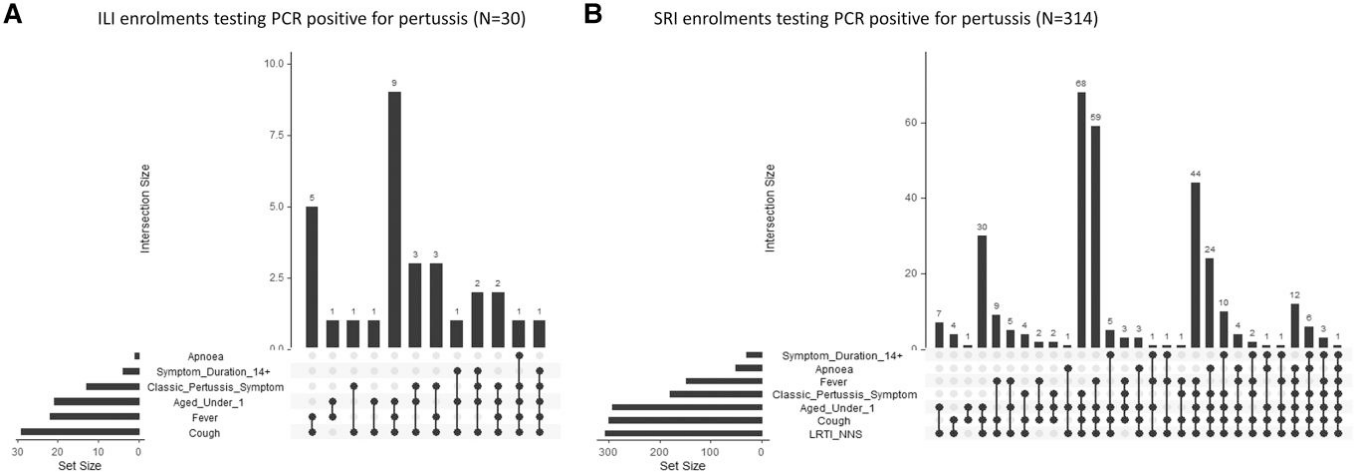
UpSetR chart to map criteria of respiratory illness surveillance case definitions in PCR-confirmed pertussis cases among children aged <5 years enrolled in (*A*) ILI outpatient surveillance (n = 30) and (*B*) SRI hospital-based surveillance (n = 314). Set size horizontal bars on the left indicate the number of cases with the relevant symptom or aspect of case definition. Intersection vertical bars at the top of plot represent the size of intersecting symptom sets. Black dots indicate which symptoms or aspects are included in an intersection. Lines connecting dots indicate which combinations of symptoms or aspects of case definitions are involved in a set. Apnea is included in the modified WHO pertussis case definition as well as the WHO case definition. Symptom duration >14 days is part of the WHO pertussis case definition. Classic pertussis symptom refers to paroxysmal coughing, posttussive vomiting, or an inspiratory whoop and is included in the modified WHO pertussis case definition as well as the WHO pertussis case definition. Age <1 year is an aspect in the modified WHO pertussis case definition as well as the WHO case definition. Fever is a symptom in the ILI and WHO SARI case definitions. Cough is a symptom in the ILI and WHO SARI case definitions, the modified WHO pertussis case definition, as well as the WHO pertussis case definition. LRTI/NNS is a clinician diagnosis included in the SRI case definition. ILI, influenza-like illness; LRTI, lower respiratory tract infection; NNS, neonatal sepsis; PCR, polymerase chain reaction; SARI, severe acute respiratory infection; SRI, severe respiratory illness; WHO, World Health Organization.

Among the 30 PCR-confirmed pertussis cases, 9 (30.0%) were aged <1 year and presented with a cough and fever, representing the most common combination of case definition criteria in ILI (Figure 3*A*). Among the 314 PCR-confirmed pertussis cases, 68 (21.7%) were aged <1 year and had clinician-diagnosed neonatal sepsis or lower respiratory tract infection and presented with cough and 1 of the classic pertussis symptoms (posttussive vomiting or inspiratory whoop), representing the most common combination of case definition criteria in participants with SRI (Figure 3*B*).

## DISCUSSION

In this study, *B pertussis* infection was confirmed in 0.7% of enrolled outpatients with ILI and 1.6% of hospitalized patients with SRI, with distinct peaks in summer (December) and a marked reduction during the COVID-19 pandemic. The WHO pertussis case definition demonstrated low sensitivity, missing 44.3% of laboratory-confirmed pertussis cases in SRI. Although the modified WHO pertussis case definition—including apnea and omitting the cough duration criterion—improved sensitivity, it still missed 39.8% of cases. Both definitions had high specificity, indicating that children not meeting either case definition were likely true pertussis negatives. These findings highlight the limitations of current pertussis case definitions and support the need to review the WHO pertussis case definition to better balance sensitivity and specificity, considering the local epidemiology and clinical presentation across ages [31].

Sensitivity for the modified WHO pertussis case definition in our study (60%) was lower than that in a previous South African study (84%), possibly due to differences in design, period, and population [16]. Nonetheless, our findings support that including apnea and omitting cough duration improves sensitivity but reduces specificity [16, 17], although many PCR-confirmed pertussis cases—similar to the WHO pertussis case definition—were still missed. A large proportion of children identified by the surveillance case definitions were false positives (>90%), reflecting the low prevalence of pertussis in this population and the resulting low PPV values, while NPV values remained high. In this surveillance, pertussis-specific treatment was given only to PCR-confirmed *B pertussis* cases; however, in routine clinical practice, relying on the case definitions alone could result in unnecessary treatment. Patients with PCR-positive SRI were younger than patients with PCR-positive ILI. In addition, patients with PCR-positive ILI had milder, atypical presentations, whereas patients with PCR-positive SRI presented with classic pertussis symptoms, such as apnea, paroxysmal cough, and posttussive vomiting, resulting in higher sensitivity for the WHO and modified pertussis definitions. These findings highlight the variability in clinical presentations of pertussis among children aged <5 years, emphasizing the need for age-specific surveillance definitions, which would be of great value in clinical settings to ensure timely pertussis-specific treatment, the prophylaxis of close and vulnerable contacts, and the prevention severe outcomes in infants. Notably, the median age of hospitalized *B pertussis*–positive cases was 1.7 months—an age group too young to be fully protected by routine infant vaccination, which begins at 6 weeks, with protection expected only 2 weeks after the first dose. The recent introduction of maternal pertussis vaccination in SA (2024) is therefore a necessary intervention, and understanding how case definitions perform will ensure accurate detection and reliable surveillance to support the upcoming evaluation of maternal pertussis vaccine effectiveness.

A strength of this research is the long-term, systematically collected surveillance data across diverse provinces and health care settings. Despite not having sentinel sites in all provinces of SA, the data reliably reflect national trends and epidemiology [32]. A notable limitation of this study is the potential misclassification of true *B pertussis*–positive cases as negative due to several factors: (1) using a Ct cutoff <35 for IS*481*-positive specimens, as the clinical relevance of higher Ct values is unclear and may indicate residual DNA or atypical cases [8]; (2) incomplete symptom recognition or recall; and (3) the collection of nasopharyngeal swabs instead of induced sputum, which have a higher yield for *B pertussis* detection but are not feasible for routine systematic surveillance [7, 8, 23]. These factors likely led to an underestimation of *B pertussis* prevalence, which was considerably lower than the 5% expected during sample size calculation and may have affected the precision of estimates. The lower-than-expected prevalence may also be due to changes in *B pertussis* epidemiology or differences in surveillance settings and populations. Some characteristics of case definitions were based on physician diagnosis and admitting practices, which may vary by clinician and site. The analysis was also limited to cases with a valid PCR result, although the small proportion of missing laboratory tests (ILI: 1.6%, 66/4191; SRI: 0.9%, 181/19 696) is unlikely to have introduced bias. It is important to acknowledge that while PCR-positive *B pertussis* cases may occasionally reflect asymptomatic infection or coinfection with other pathogens, studies in SA report high attributable fractions (>90%) among confirmed cases, suggesting that most detections in our setting represent true pertussis and are clinically relevant [7, 8].

Increased sensitivity with balanced specificity of surveillance case definitions to detect *B pertussis* is necessary to ensure a timeous response given the increasing incidence of pertussis and the return of cyclical peaks of infection. Challenges with case definition performance have been observed for other respiratory pathogens, such as respiratory syncytial virus, where (1) standard severe acute respiratory infection definitions requiring fever underestimated disease burden, particularly in infants aged <6 months, and (2) refining definitions to remove the fever requirement improved detection and helped inform public health interventions [18, 33, 34]. Therefore, we recommend revising the WHO pertussis case definition, with attention to age-specific presentations of pertussis, to improve sensitivity while maintaining specificity. In the meantime, integrating surveillance of *B pertussis* with syndromic surveillance of respiratory pathogens, as demonstrated in this study and in alignment with WHO guidelines, may enhance the detection of *B pertussis* in cases that do not present with the classical symptoms while minimizing the burden on resources. Implementation would require (1) expanding screening and enrollment criteria to include pertussis-specific and other respiratory surveillance case definitions, (2) applying uniform molecular testing for all enrollments, and (3) amending case report forms to incorporate pertussis-specific symptoms (eg, posttussive vomiting, paroxysmal cough, and whoop) and pertussis vaccination status, including maternal vaccination status for infants. Although testing all ILI and SRI enrollments for *B pertussis* increases sensitivity, the specificity is low (<10%), and cost implications are a consideration. Nonetheless, this strategy leverages existing surveillance infrastructure and multiplex platforms. Further research is needed to better understand *B pertussis* clinical presentations and the factors associated with positive test results that fail to meet the pertussis surveillance case definitions, to improve detection and more accurately estimate the burden of pertussis in SA.

The WHO and modified pertussis case definitions missed many laboratory-confirmed pertussis cases and likely led to an underestimation of the true disease burden of pertussis in settings relying only on these definitions. Revising the WHO pertussis case definition and integrating pertussis into broader syndromic surveillance may improve detection and understanding of the burden of pertussis, particularly in young infants to guide public health policy.

## Supplementary Material

jiaf501_Supplementary_Data

## Supplementary Data

Supplementary materials are available at *The Journal of Infectious Diseases* online (http://jid.oxfordjournals.org/). Supplementary materials consist of data provided by the author that are published to benefit the reader. The posted materials are not copyedited. The contents of all supplementary data are the sole responsibility of the authors. Questions or messages regarding errors should be addressed to the author.
