# Supplementary material for: Comparing Respiratory Illness Surveillance Case Definitions to Detect Bordetella pertussis in Children Aged <5 Years With Respiratory Illness in South Africa, 2017–2023
Source: J Infect Dis. 2025 Oct 6;233(1):e34–43. doi: 10.1093/infdis/jiaf501 (PMC12811878; doi:10.1093/infdis/jiaf501)
Supplement: jiaf501_Supplementary_Data [file jiaf501_supplementary_data.zip › Supplementary_material.docx]

**Supplementary material:**

Table 1: Influenza-like illness (ILI) and severe respiratory illness (SRI) inclusion and exclusion criteria

| ILI |
| --- |
| **Inclusion criteria:** |
| 1. Patients attending the clinic (Monday-Friday) and meeting the study case definitions will be eligible for inclusion 2. Patients/ guardians who refuse HIV testing are still included |
| **Exclusion criteria:** |
| 1. Patients who reside outside of the catchment areas |
| SRI |
| **Inclusion criteria** |
| 1. Patients admitted to the surveillance site and meeting the study case definitions will be eligible for inclusion 2. Sample collection must be within 48 hours of admission 3. Patients admitted from 17H00 on Sunday until 13H00 on Friday are eligible for enrolment 4. Patients/ guardians who refuse HIV testing are still included |
| **Exclusion criteria:** |
| 1. Transfers directly to ICU from other hospitals and not passing through the admissions ward 2. New-borns who have not been discharged following delivery 3. Any patient who was previously enrolled in SRI less than one month ago 4. Nosocomial sepsis/ lower respiratory tract infection (LRTI) if patient was readmitted within 7 days 5. Any patient/ guardian who declines 6. Out of catchment |

Table 2: Facility name and province of Influenza-Like Illness (ILI) and Severe Respiratory Illness (SRI) sentinel surveillance sites enrolling participants aged <5 years.

| Facility | Province | ILI/ SRI |
| --- | --- | --- |
| Agincourt Clinic | Mpumalanga | ILI |
| Eastridge Clinic | Western Cape | ILI |
| Harry Gwala Gateway Clinic | KwaZulu-Natal | ILI |
| Harry Gwala Hospital | KwaZulu-Natal | SRI |
| Jouberton Clinic | North West | ILI |
| Klerksdorp Hospital | North West | SRI |
| Mapulaneng Hospital | Mpumalanga | SRI |
| Matikwane Hospital | Mpumalanga | SRI |
| Mitchell's Plain Hospital | Western Cape | SRI |
| Red Cross War Memorial Children's Hospital | Western Cape | SRI |
| Rahima Moosa Hospital | Gauteng | SRI |
| Tintswalo Hospital | Mpumalanga | SRI |

**Abbreviations**: ILI: influenza-like illness; SRI: severe respiratory illness

Figure 1: Flowchart of patients aged <5 years enrolled in the influenza-like illness (ILI) and severe respiratory illness (SRI) programmes, South Africa, 2017-2023. Abbreviations: ILI: influenza-like illness; SRI: severe respiratory illness; PCR: polymerase chain reaction; *B. pertussis: Bordetella pertussis.*


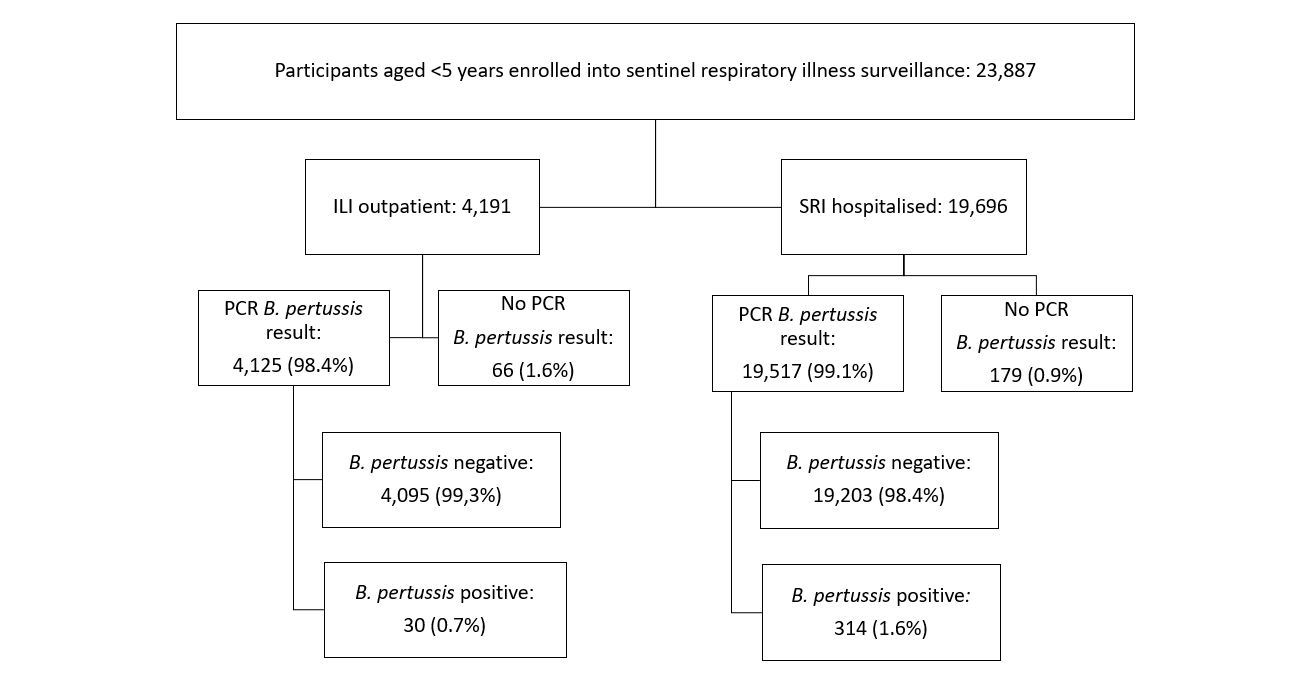


Table 3. Comparison of children aged <5 years seeking healthcare for respiratory illness by *Bordetella pertussis* laboratory test result, identified through influenza-like illness surveillance, and severe respiratory illness sentinel surveillance, South Africa, 2017–2023 (N=23,642).

|  | ILI sentinel surveillance, N=4,125 | | | | | | SRI sentinel surveillance, N=19,517 | | | | | |
| --- | --- | --- | --- | --- | --- | --- | --- | --- | --- | --- | --- | --- |
| Characteristic | Total tested | Negative N=4,095 | | Positive N=30 | | P-value | Total tested | Negative N=19,203 | | Positive N=314 | | P-value |
|  |  | n | (%) | n | (%) |  |  | n | (%) | n | (%) |  |
| **Age Group:** |  |  |  |  |  | **<0.001** |  |  |  |  |  | **<0.001** |
| < 3 months | 261 | 249 | (6.1) | 12 | (40.0) |  | 6,672 | 6,417 | (33.4) | 255 | (81.2) |  |
| 3 – 11 months | 1,166 | 1,157 | (28.3) | 9 | (30.0) |  | 6,516 | 6,478 | (33.7) | 38 | (12.1) |  |
| 1 – 4 years | 2,698 | 2,689 | (65.7) | 9 | (30.0) |  | 6,329 | 6,308 | (32.9) | 21 | (6.7) |  |
| **Clinical criteria:** |  |  |  |  |  |  |  |  |  |  |  |  |
| Apnoea (aged <1 year) | 7 | 6 | (0.4) | 1 | (4.8) | **0.005** | 593 | 541 | (4.2) | 52 | (17.8) | **<0.001** |
| Cough | 4,067 | 4,038 | (98.6) | 29 | (96.7) | 0.358 | 16,954 | 16,654 | (87.2) | 300 | (96.2) | **<0.001** |
| Paroxysmal cough | 675 | 664 | (16.2) | 11 | (36.7) | **0.003** | 2,228 | 2,093 | (10.9) | 135 | (43.0) | **<0.001** |
| Posttussive vomit | 724 | 715 | (17.5) | 9 | (30.0) | 0.072 | 2,135 | 2,033 | (10.7) | 102 | (32.8) | **<0.001** |
| Whoop | 110 | 110 | (2.7) | 4 | (13.3) | **<0.001** | 428 | 379 | (2.0) | 49 | (15.6) | **<0.001** |
| History of fever | 3,862 | 3,840 | (93.8) | 22 | (73.3) | **<0.001** | 12,657 | 12,509 | (65.2) | 148 | (47.1) | **<0.001** |
| **Duration of symptoms:** |  |  |  |  |  | **<0.001** |  |  |  |  |  | **<0.001** |
| ≤10 days | 108 | 4,002 | (97.5) | 25 | (86.2) |  | 18,712 | 18,437 | (96.1) | 275 | (87.9) |  |
| >10 days | 108 | 104 | (2.6) | 4 | (13.8) |  | 796 | 758 | (4.0) | 38 | (12.1) |  |
| **Risk factors:** |  |  |  |  |  |  |  |  |  |  |  |  |
| Prematurity | 193 | 193 | (4.7) | 0 | (0.0) | 0.223 | 2,675 | 2,633 | (13.7) | 42 | (13.4) | 0.864 |
| Malnutrition | 268 | 267 | (6.6) | 1 | (3.3) | 0.468 | 3,490 | 3,432 | (18.1) | 58 | (18.6) | 0.806 |
| Comorbidity | 18 | 18 | (0.4) | 0 | (0.0) | 0.716 | 893 | 884 | (4.6) | 9 | (2.9) | 0.144 |
| Vaccination: |  |  |  |  |  | **<0.001** |  |  |  |  |  | **<0.001** |
| No vaccination | 206 | 199 | (6.4) | 7 | (29.2) |  | 5,038 | 4,846 | (29.4) | 192 | (66.0) |  |
| 1 primary dose | 121 | 117 | (3.8) | 4 | (16.7) |  | 1,763 | 1,699 | (10.3) | 64 | (22.0) |  |
| 2 primary doses | 170 | 170 | (5.4) | 0 | (0.0) |  | 1,230 | 1,219 | (7.4) | 11 | (3.8) |  |
| 3 primary doses | 2,651 | 2,638 | (84.4) | 13 | (54.2) |  | 8,735 | 8,711 | (52.9) | 24 | (8.3) |  |
| HIV status: |  |  |  |  |  | **0.005** |  |  |  |  |  | 0.264 |
| HIV unexposed uninfected | 3,860 | 3,833 | (96.8) | 27 | (90.0) |  | 16,697 | 16,426 | (91.5) | 271 | (92.8) |  |
| HIV exposed uninfected | 118 | 116 | (2.9) | 2 | (6.7) |  | 1,382 | 1,361 | (7.6) | 21 | (7.2) |  |
| HIV exposed infected | 12 | 11 | (0.3) | 1 | (3.3) |  | 157 | 157 | (0.9) | 0 | (0.0) |  |

**Note:** Fever: recorded ≥38°C or reported on history. Included in comorbidity: chronic lung, neurological, heart, kidney, liver, and immune conditions; diabetes; stroke; burns; obesity; and other congenital disorders. Considered premature if gestational age <37 weeks. Considered vaccinated with 1 primary dose if aged ≥8 weeks and received 1 vaccine dose. Considered vaccinated with 2 primary doses if aged ≥12 weeks and received 2 vaccine doses. Considered vaccinated with 3 primary doses if aged ≥16 weeks and received 3 vaccine doses. Denominator data excluded unknown responses. Bold indicates a significant p-value. **Abbreviations:** ILI: influenza-like illness; SRI: severe respiratory illness.
