# Supplementary figures and images for: Comparing Respiratory Illness Surveillance Case Definitions to Detect Bordetella pertussis in Children Aged <5 Years With Respiratory Illness in South Africa, 2017–2023
Source: J Infect Dis. 2025 Oct 6;233(1):e34–43. doi: 10.1093/infdis/jiaf501 (PMC12811878; doi:10.1093/infdis/jiaf501)

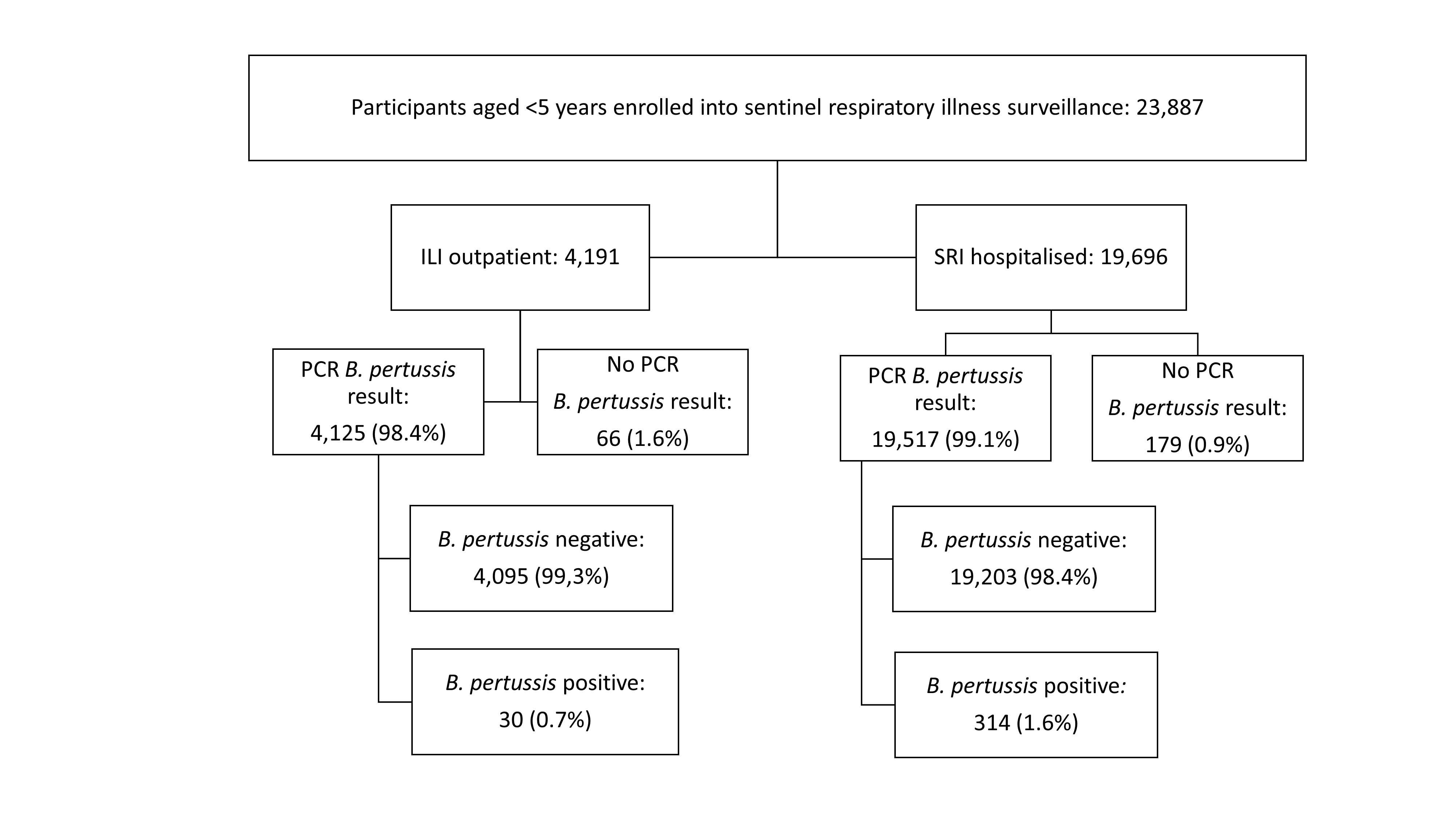

Supplement: jiaf501_Supplementary_Data [file jiaf501_supplementary_data.zip › supplementary_material_Figure_1.tif]
